# Neuroprotective and Neurotoxic Effects of Glial-Derived Exosomes

**DOI:** 10.3389/fncel.2022.920686

**Published:** 2022-06-22

**Authors:** Karina Oyarce, María Yamila Cepeda, Raúl Lagos, Camila Garrido, Ana María Vega-Letter, María Garcia-Robles, Patricia Luz-Crawford, Roberto Elizondo-Vega

**Affiliations:** ^1^Laboratorio de Neuroinmunología, Facultad de Medicina y Ciencia, Universidad San Sebastián, Concepción, Chile; ^2^Laboratorio de Biología Celular, Departamento de Biología Celular, Facultad de Ciencias Biológicas, Universidad de Concepción, Concepción, Chile; ^3^Facultad de Medicina, Centro de Investigación Biomédica, Universidad de los Andes, Santiago, Chile

**Keywords:** exosomes, astrocytes, microglia, oligodendrocyte, neurotoxic, neuroprotective, neuroinflammation

## Abstract

Exosomes derived from glial cells such as astrocytes, microglia, and oligodendrocytes can modulate cell communication in the brain and exert protective or neurotoxic effects on neurons, depending on the environmental context upon their release. Their isolation, characterization, and analysis under different conditions *in vitro*, in animal models and samples derived from patients has allowed to define the participation of other molecular mechanisms behind neuroinflammation and neurodegeneration spreading, and to propose their use as a potential diagnostic tool. Moreover, the discovery of specific molecular cargos, such as cytokines, membrane-bound and soluble proteins (neurotrophic factors, growth factors, misfolded proteins), miRNA and long-non-coding RNA, that are enriched in glial-derived exosomes with neuroprotective or damaging effects, or their inhibitors can now be tested as therapeutic tools. In this review we summarize the state of the art on how exosomes secretion by glia can affect neurons and other glia from the central nervous system in the context of neurodegeneration and neuroinflammation, but also, on how specific stress stimuli and pathological conditions can change the levels of exosome secretion and their properties.

## Introduction

Exosomes have been largely studied for their role in cell-to-cell communication and their potential to modulate cellular function, through direct transfer of metabolites and proteins, or through gene regulation by miRNA and long non-coding RNAs transfer (Jin et al., 2021; Rastogi et al., 2021). They are a subtype of a larger group of extracellular vesicles (EVs) constituted also of apoptotic bodies and microvesicles (smVs) (Mathieu et al., 2019). Exosomes originate after the fusion of multivesicular bodies with the plasma membrane and the later release of intraluminal vesicles, reaching sizes between 30 and 150 nm. Although most studies on exosomes report their isolation within this size fraction through differential centrifugation, it is important to note that some smVs that originate by direct budding from plasma membrane might also be present in this fraction, because of their size ranges from 40 nm to 1 μM (Mathieu et al., 2019; Rastogi et al., 2021). For this reason, some literature uses the term EV and exosome interchangeably. For a comprehensive review regarding their formation, isolation, and analysis, readers can refer to Rastogi et al. (2021).

Exosomes have broad therapeutic advantages, such as low immunogenicity, convenient storing, and high biosafety (Marote et al., 2016). They are currently being proposed as excellent biomarkers for diagnosing several brain diseases, as they can be recovered and screened from plasma and cerebrospinal fluid (CSF) from patients (Liu et al., 2019; Shaimardanova et al., 2020; Rastogi et al., 2021). However, to better understand how illness progresses and to provide potential therapeutic strategies based on the information of these exosomes, it is necessary to determine the cell type from which they originate.

Different strategies have been used for identifying the presence of glial-derived exosomes in body fluids, such as plasma and CSF. Some of them have evaluated the presence of GFAP (for astrocyte origin) or isolectin B4 (IB4) (for microglial origin) in total purified exosomes by either western blot of flow cytometry (Joshi et al., 2014; Willis et al., 2017; Kodidela et al., 2020; Mondello et al., 2020; Wang et al., 2021). Others have done proteomic analysis, finding GFAP expression as well (Manek et al., 2018), while some have identified glial exosomes by ELISA sandwich, combining glutamate aspartate transporter (GLAST) and CD81 in plasma samples (Ohmichi et al., 2019).

**FIGURE 1:**
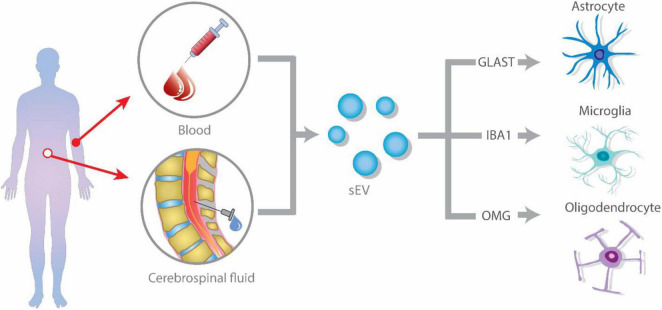
Glial-derived exosomes isolation strategies from blood or plasma. Extracellular vesicles (EVs) are released by glial cells, containing different cargo molecules such as cytokines, proteins, and non-coding RNA. The ability of EVs to cross the blood–brain barrier (BBB), allows them to enter the peripheral blood and cerebrospinal fluid (CSF). Isolation of glial cell-derived exosomes from blood or CSF is accomplished by identifying glial-specific proteins surface markers, such as glutamate aspartate transporter (GLAST) for astrocytes, CD11b and isolectin B4 (IB4) for microglia, and oligodendrocyte-myelin glycoprotein (OMG) for oligodendrocytes.

However, fewer studies have specifically isolated glial-derived exosomes to further characterize their content or to conduct functional assays. In these studies, astrocyte-derived exosomes are purified by using biotinylated antibodies against GLAST followed by streptavidin-agarose resin chromatography (Goetzl et al., 2016) or by magnetic beads conjugated with GLAST antibodies and further FACS (Winston et al., 2019), while microglia-derived exosomes are purified by magnetic beads conjugated with Tmem119 and further FACS (Cohn et al., 2021; Kumar et al., 2021). So to study glial-derived exosomes, researchers have mostly rely on the use of classical cell lines like BV2 and M9 (microglia), OliNeu (oligodendrocytes), or primary microglia, and astrocytes cultures (Figure 1).

**FIGURE 2:**
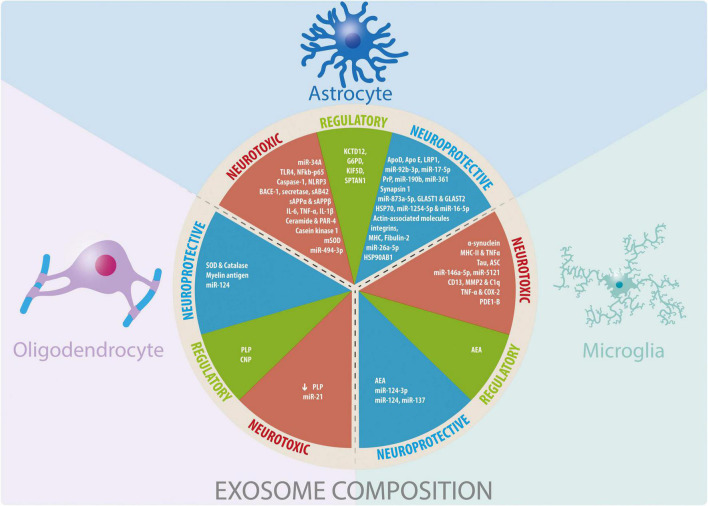
Glial cells-derived exosomes coposition and their participation in neuroprotective, neurotoxic, and regulatory functions. Summary of the information reported about the cargo of exosomes derived from glial cells and their effects observed in different physiological and pathophysiological models *in vitro* and *in vivo*.

In this review we summarize evidence on how exosomes secretion by glia can affect neurons and other glia from the central nervous system in the context of pathological conditions associated with neuroinflammation and neurodegeneration. We also address how different cell stressors can change exosomes properties. It should be noted that for this review we have only considered studies that report an increase in exosomes released by glial cells assessing their beneficial or detrimental effect (Figure 2).

## Astrocyte-Derived Exosomes

### Their Detrimental Effects

Several studies show that exosomes from astrocytes can participate in the pathophysiology of neurodegenerative diseases, as well as cerebrovascular pathologies (Table 1). Depending on their cargo they can have neurotoxic effects due to the presence of high levels of glutamate, viral proteins, misfolded proteins, metalloproteinases, NO, arachidonic acid, and pro-inflammatory cytokines. For instance, when astrocytes are treated with lipopolysaccharide (LPS), their exosomes are enriched in miR-34A, increasing the vulnerability of dopaminergic neurons to mitochondrial complex inhibitors drugs (e.g., MPP^+^ and 6-OHDA) both *in vitro* and *in vivo* (Mao et al., 2015).

**TABLE 1:** Summary of neuroprotective and neurotoxic effects of astrocytes-derived exosomes with identified cargo.

Role	Stimuli/disease	Cargo	Effect observed	References
	LPS	miR-34A	Increases susceptibility to mitochondrial damage	Mao et al., 2015
	Ethanol	TLR4, NF-κb-p65, IL1R, caspasa-1, NLRP3	Amplifies neuroinflammatory response *via* TLR4	Ibanez et al., 2019
	AD	BACE-1, secretase, sAB42, sAPPα y sAPPβ	Contribution to AB spreading?	Goetzl et al., 2016
	AD	IL-6, TNF-α, IL1β, and some complement proteins	Amplifies neuroinflammatory response	Goetzl et al., 2018
	Aβ	Ceramide and PAR-4	Glial apoptosis and neurodegeneration	Wang et al., 2012
Neurotoxic	IL-1β	Casein kinase 1	Promoters Aβ synthesis in neurons	Li et al., 2020
	Overexpression of mutated SOD1	Mutated SOD1	Reduces the viability of spinal neurons	Silverman et al., 2019
	ALS	miR-494-3p	Alteration of neurite length in motoneurons	Varcianna et al., 2019
Neuroprotective	–	ApoD	Promotes survival and functional integrity under oxidative stress	Pascua-Maestro et al., 2019
	OGD	miR-92b-3p	Reduces neuronal death induced by OGD	Xu et al., 2019
	OGD	PrP	Reduces neuronal death due to H_2_O_2_ oxidative stress	Guitart et al., 2016
	–	miR-190b	Reduces autophagy and neuronal apoptosis induced by OGD	Pei et al., 2020
	OGD + H_2_O_2_ + KCl	Synapsin 1	Promotes cell survival and neurite growth under oxidative stress	Wang et al., 2011
	TBE	miR-873a-5p	Promotes anti-inflammatory phenotype in microglia	Long et al., 2020
	PMA	GLAST 1 and GLAST 2	Reduces neurotoxicity induced by glutamate?	Gosselin et al., 2013
	Thermal stress	HSP70	Reduces neuroinflammation and misfolded protein aggregation?	Taylor et al., 2007
	IL-1β and TNF-α	miR-1254-5p and miR-16-5p	Reduces dendritic complexity in hippocampal neurons	Chaudhuri et al., 2018
	IL-1β	Actin-associated molecules, integrins, MHC	Reduces neuronal branching and firing rate	You et al., 2020
	–	Fibulin-2	Stimulates dendritic spine formation and synapse	Patel and Weaver, 2021
	Aldolase C overexpression	miR-26a-5p	Reduces dendritic complexity in developing hippocampal neurons	Luarte et al., 2020
	–		Reduces infarct size, neuronal death, neurological damage, and inflammation	
	–	miR-361	Negatively regulates neuronal apoptosis	Bu et al., 2020
	–	miR-17-5p		Du et al., 2021
		HSP90AB1, LRP1, ApoE		Chun et al., 2021
Regulatory	–	KCTD12, G6PD, KIF5D, SPTAN1	Regulates neuronal excitability	Chun et al., 2021

Most of the data regarding neurotoxic effects of astrocytes-derived exosomes are associated with chronic inflammation in models of Alzheimer’s disease (AD), ethanol exposure, and amyotrophic lateral sclerosis (ALS). In a model of ethanol-induced neuroinflammation, where glial activation through Toll-like receptor 4 (TLR4) has been previously observed (Blanco et al., 2005; Fernandez-Lizarbe et al., 2009), ethanol stimulated the production of EVs from primary mice cortical astrocytes in culture, which contained higher levels of pro inflammatory molecules, such as TLR4, NF-κB-p65, IL-1R, caspase-1, NOD-like receptor 3 (NLRP3). Importantly, astrocytes knock-out for TLR4 did not respond to ethanol. In addition, these exosomes were internalized by cortical neurons *in vitro*, which responded by elevating cyclooxygenase 2 (Cox2) levels, suggesting EVs can amplify the neuroinflammatory response, in a manner dependent on TLR4 activation (Ibanez et al., 2019).

Both AD murine models and human samples from AD patients suggests a detrimental role of exosomes derived from astrocytes. Astrocyte-derived exosomes isolated from the plasma of AD patients through biotinylated antibodies against GLAST, exhibit enrichment of β-site amyloid precursor protein-cleaving enzyme 1 (BACE-1), γ-secretase, soluble Aβ42, soluble amyloid precursor protein (sAPPα and sAPPβ), all these crucial for the maintenance of Aβ42 production machinery (Goetzl et al., 2016). In addition, these exosomes have high levels of interleukin 6 (IL-6), TNF-α, and IL-1β, as well as several complement proteins, suggesting that the pathogenic role of astrocytes in AD might be in part caused by the secretion of exosomes with inflammatory properties (Goetzl et al., 2018).

In another study, Aβ peptide activates sphingomyelinase-2 in primary astrocytes culture, which increases the release of pro-apoptotic exosomes enriched in ceramide and prostate apoptosis response-4 protein (PAR-4), suggesting these exosomes might contribute to glial apoptosis and neurodegeneration seen in AD (Wang et al., 2012). Interestingly, *in vivo* inhibition of sphingomyelinase-2 through GW4869, decreases amyloid plaques, ceramides, and exosomes in brain tissue and serum, suggesting that pharmacological inhibition of sphingomyelinase-2 could be a potential treatment for AD, by decreasing brain exosomes with neurotoxic effect (Dinkins et al., 2014). Moreover, when neuroglia co-cultures enriched in astrocytes are exposed to Aβ42, they release exosomes that induce neuronal cell death by apoptosis (Beretta et al., 2020).

Interestingly, when astrocytes are treated with IL-1β, they release exosomes enriched in casein kinase 1, which promotes Aβ synthesis in hippocampal neurons *in vitro*, through a mechanism depending on GSK3-β/β-catenin (Li et al., 2020).

In ALS there is also evidence pointing toward astrocyte derived exosomes as contributors for disseminating the disease. For instance, it has been observed that exosomes from astrocytes that overexpress a mutated form of superoxide dismutase 1 (SOD1) transfer this altered protein to spinal neurons in culture, reducing their viability (Silverman et al., 2019).

Exosomes from induced astrocytes in culture, deriving from ALS patient’s fibroblast and harboring specific mutation C9orf72, have neurotoxic effect on motoneuron *in vitro*, also altering their neurite length (Varcianna et al., 2019). This could be explained by the lower levels of miR-494-3p in these exosomes, which is also diminished in cortico-spinal tracts from postmortem ALS biopsies (Varcianna et al., 2019).

### Their Beneficial Effects

On the other hand, several other studies have shown that astrocytes-derived exosomes can have neuroprotective properties against oxidative stress, ischemia and nutrient deprivation, potentiating cell survival, and neurite growth (Upadhya et al., 2020) (Table 1). In general terms, exosomes derived from both human and mice astrocytes are enriched in apolipoprotein D (ApoD), a molecule described for its beneficial properties against aging, AD, and multiple sclerosis (Dassati et al., 2014; Li et al., 2015; Navarro et al., 2018). Also, these exosomes can transfer ApoD into neurons, promoting their survival and functional integrity after paraquat-induced oxidative stress (Pascua-Maestro et al., 2019). Other proteins with neuroprotective functions have also been described to be present in exosomes derived from rat astrocytes cultures under non-inflammatory conditions. Some of them has been associated with neurogenesis, angiogenesis, neuronal plasticity, and protection against oxidative stress (Matsuzaki et al., 2001; Svensson et al., 2002; Lange et al., 2016; Shim and Madsen, 2018; Froger et al., 2020), such as neuroglobin (Venturini et al., 2019), fibroblast growth factor-2 (FGF-2), and vascular endothelial growth factor (VEGF) (Proia et al., 2008). In human astrocyte-derived exosomes, other proteins with neuroprotective effects have been identified by proteomic analysis, such as heat shock protein 90 alpha family class B member 1 (HSP90AB1), lipoprotein receptor-related protein 1 (LRP1), and apolipoprotein E (APOE), which negatively regulates neuronal apoptosis. In addition, these exosomes contain proteins that regulate neuronal excitability such as potassium channel tetramerization domain containing 12 (KCTD12), glucose-6-phosphate dehydrogenase (G6PD), kinesin family member 5B (KIF5B), and spectrin-alpha non-erythrocytic 1 (SPTAN1) (Chun et al., 2021).

Exosomes from astrocytes cultured under glucose deprivation and hypoxic conditions (OGD) are enriched in miR-92b-3p (Xu et al., 2019) and PrP (Guitart et al., 2016) a surface glycoprotein normally involved in oxidative stress sensing (Onodera et al., 2014). When exosomes were incorporated by primary neurons cultures under OGD, they reduced neuronal death (Xu et al., 2019) and H_2_O_2_ oxidative stress (Guitart et al., 2016). Related to PrP presence in exosomes, another study previously showed that exosomes from astrocytes also contain stress-inducible protein 1 (STI1), which has a great affinity for PrP (Hajj et al., 2013), that could suggest STI1 is relevant for PrP incorporation into exosomes. However, misfolding of PrP causes infectious spongiform encephalopathy, so it would be interesting to address whether astrocyte derived exosomes could also lead to prionic disease transmission or not.

Another study on primary astrocytes enriched in miR-190b also shows a protective effect when added to the hippocampal cell line HT-229 under OGD (Pei et al., 2020), by targeting autophagy-related gene 7 (Atg7), which reduces autophagy and neuronal apoptosis.

Other studies have shown that when astrocytes are cultured under OGD, H_2_O_2_, and KCl, they secrete synapsin 1-enriched exosomes. Synapsin 1 promotes cell survival and neurite growth in hippocampal neurons cultured under oxidative stress (Wang et al., 2011), having already been described as a neuroprotective protein (Upadhya et al., 2020). Although the authors do not prove direct transfer of synapsin 1 from astrocytes exosomes to neurons, they suggest that exosome-mediated transfer of synapsin 1 could be a relevant mechanism for neuroprotection.

In addition to these studies, in rat models of ischemic injury, administration of astrocyte-derived exosomes before or after hypoxic damage reduced the infarct size, inflammation, neuronal death, and associated neurological damage, partly due to enrichment of miR-17-5p (Du et al., 2021) or miR-361 (Bu et al., 2020), respectively. Particularly for miR-17-5p, the authors showed *in vitro* that one of its targets is BCL2 interacting protein 2 (BNIP2), a pro-apoptotic regulator.

On the other hand, astrocytes cultured with brain extracts from mice with traumatic brain encephalopathy (TBE) release more exosomes than non-stimulated astrocytes, and in addition these exosomes promote more M2 polarization of microglial cultures. A miRNA expression analysis revealed enrichment of miR-873a-5p and the authors show that overexpression of this miRNA directly on microglia, increases IL-4, and arginase-1, while decreases NF-kB signaling, thus promoting anti-inflammatory phenotype (Long et al., 2020). Moreover, astrocyte exosomes can promote recovery of TBI-like injured neurons when cultured in a transwell system, reducing apoptosis rate, and increasing mitochondrial function (Chen et al., 2020). When these astrocyte exosomes are injected into the lateral ventricles of rats subjected to TBI, brain damage recovery can be observed after a week (Chen et al., 2020).

Another study showed that when rat astrocytes are treated with phorbol-12-myristate 13-acetate (PMA), as well as spinal cord explants from rats suffering spared nerve injury, they release EV containing GLAST 1 and 2 (Gosselin et al., 2013), known to reduce neurotoxicity induced by glutamate release in neurodegenerative diseases such as Parkinson’s disease (PD), AD, ALS, and Huntington’s disease (Iovino et al., 2020; Todd and Hardingham, 2020). Also, astrocytes from chick spinal cord cultures submitted to thermal stress, release exosomes containing heat shock protein HSP70 (Taylor et al., 2007), which is known to interfere with apoptosis and inflammatory signaling, in addition to a reduced oxidative damage, by preventing protein misfolding and aggregation (Beere and Green, 2001; Vabulas et al., 2002; Tidwell et al., 2004; Novoselova et al., 2005).

Altogether this evidence shows a protective effect of astrocytes-derived exosomes against different types of stress, which could be used in therapies for neurodegenerative diseases.

On the other hand, evidence shows that exosomes from astrocytes can modulate neurite outgrowth. For instance, exosomes from astrocytes primed with IL-1β and TNF-α, which increase their content on miR-125a-5p and miR-16-5p, reduce dendritic complexity in hippocampal neurons (Chaudhuri et al., 2018). This effect can be abrogated with antisense oligonucleotide inhibitors directed against these miR. Another study has shown that astrocyte priming with IL-1β alone is able to change the proteinic cargo of exosomes, by increasing the levels of actin-associated proteins, integrins, and major histocompatibility complex (MHC). These exosomes from primed astrocytes reduce neuronal branching and firing rate (You et al., 2020). On the contrary, when astrocytes are stimulated with exosomes from mesenchymal stem cells that overexpress miR-133b (considered a neuroprotective miR), they increase exosome release, which increases neurite branching and elongation on cortical neurons (Xin et al., 2017).

Interestingly, a recent report with astrocyte exosomes under physiological conditions, showed that they stimulate dendritic spine formation and synapsis in cortical neurons by TGF-β activation. This effect was mediated by the presence of fibulin-2 in the astrocyte exosomes (Patel and Weaver, 2021).

However, another study using exosomes from astrocytes obtained after *in utero* electroporation of GFP, reduces dendritic complexity in developing hippocampal neurons by a mechanism that depends on miR-26a-5p (Luarte et al., 2020). This effect can be also increased if aldolase C, a glycolytic enzyme, is enriched in astrocytes exosomes. It might be necessary to address whether the opposed effects on neuronal plasticity from these newer studies are due to different neuronal types used, to different astrocytes culture conditions or to methodological interventions.

## Microglia-Derived Exosomes

### Their Contribution to the Spreading of Toxic Proteins

Evidence has shown that microglial exosomes can promote and propagate neuroinflammation, not only through delivery of pro-inflammatory cytokines or miRNA that control inflammatory and neurodegenerative pathways, but also by contributing to the spreading of toxic proteins (Guo et al., 2020) (Table 2). For instance, in the context of PD, it has been shown that exosomes isolated from CSF of PD patients positive for CD11b, which is an indicative of microglial origin, harbors higher levels of oligomeric α-synuclein, and can also propagate α-synuclein aggregation when added to neuronal cultures (Guo et al., 2020). In addition, microglia cultures treated with human preformed α-synuclein fibrils (PFF) and LPS secrete higher amounts of exosomes with higher levels of α-synuclein, and when these exosomes are injected into the striatum of mice, they increases α-synuclein phosphorylation in several brain regions, augmenting dopaminergic neuronal degeneration, lowering dopamine levels, and altering motor functions after 6 months. On the other hand, when α-synuclein PFF is injected into mice under pharmacological depletion of microglia, partial decrease in α-synuclein phosphorylation and aggregation can be observed in neurons (Guo et al., 2020). Altogether, these results highlight microglial contribution in the spreading of cytotoxic proteins through exosomes.

**TABLE 2:** Summary of neuroprotective and neurotoxic effects of microglia-derived exosomes with identified cargo.

Role	Stimuli/disease	Cargo	Effect observed	References
	PD	α-Synuclein	Increases Iba-1 expression, microglia number, and arborization	Xia et al., 2019
			Increases the aggregated form of α-synuclein in neurons	
	PFF and LPS	α-Synuclein	Increases dopaminergic neuron degeneration	Guo et al., 2020
	α-Synuclein	MHC-II and TNF-α	Increases neuronal cell death	Chang et al., 2013
	Tau and LPS or ATP	Tau	Increases tau dissemination on neurons	Asai et al., 2015; Clayton et al., 2021
	LPS and Mn^+2^	ASC	Increases pro-IL-1β and NLRP3 in microglia	Sarkar et al., 2019
	Th1 cytokines and LPS	miR-146a-5p	Reduces the expression of Syt1 and Nlg1	Prada et al., 2018
			Reduces dendritic spine density	
Neurotoxic	Stretch injury	miR-5121	Reduces the expression of synaptophysin, PSD-95, GluR-1	Zhao et al., 2021
			Reduces dendritic spine density	
	Ethanol	CD13, MMP2 and C1q	Increases neuronal cell death?	Mukherjee et al., 2020
	Cerebral ischemia	TNF-α and COX-2	Increases neuroinflammation	Gao et al., 2020
	OGD	PDE1-B	Induces neuronal cell death	Zang et al., 2020
	Brain extract from mTBI/	miR-124-3p	Reduces inflammation	Huang et al., 2018; Li et al., 2019; Ge et al., 2020
	Over-expression of miR-124-3p		Increases the expression of BDNF and neurogranin	
Neuroprotective			Increases neurite length and number of branches	
			Reduces Rela, VILIP-1, Aβ, and APP	
	IL-4	miR-124	Protects neurons from OGD induced cell death	Song et al., 2019
	IL-4	miR-137	Protects neurons from OGD induced cell death	Zhang et al., 2021
Regulatory	–	AEA	Inhibits presynaptic transmission in GABAergic neurons	Gabrielli et al., 2015

Studies on microglial cell line BV2 also show that α-synuclein treatment increases exosome secretion, as well as their levels of MHC-II molecules and TNF-α, inducing cell death of cortical neuronal cultures, partly due to TNF-α (Chang et al., 2013). It has been proposed that α-synuclein intracellular accumulation in microglia, by autophagy disruption, would be “alleviated” through exosomes release, which is used as a spreading mechanism for toxic proteins (Guo et al., 2020).

Another example of microglial exosomes involved in misfolded protein dissemination is given by studies on AD. For instance, when microglia are incubated with pre-aggregated Tau, and then activated with LPS or ATP, they release exosomes carrying ubiquitinated forms of Tau, which can later be incorporated by cortical neurons. Only when exosomes from Tau-treated microglia are injected into the dentate gyrus, Tau is detected in the granule neurons, not when Tau is injected alone (Asai et al., 2015). Tau propagation into neurons can be reduced when microglia is pharmacologically depleted *in vivo* (Asai et al., 2015; Clayton et al., 2021). These results suggest a role of microglial exosomes in Tau protein dissemination.

On the other hand, when neuronal cultures are incubated with a mixture of Aβ 1–42 and microglia-derived exosomes, an increase in intracellular calcium levels and cell death can be observed. Interestingly, this neurotoxicity is not observed with Aβ alone or with microglial exosomes alone (Joshi et al., 2014). Fractioning analysis of the toxic mixture and the Aβ soluble/aggregated forms present in it, revealed that only the soluble fraction was responsible for the neurotoxic effects, and that exposure to microglial exosomes disassembles Aβ aggregates, increasing the soluble forms and other cleavage Aβ products with neurotoxic effects. In addition, micro-vesicles from CSF of AD patients, positive for microglial marker IB4 (which are the most abundant from the whole pool) were also shown to be neurotoxic when combined with Aβ 1–42 and added to neuronal cultures (Joshi et al., 2014).

### Their Modulation by Trophic or Inflammatory Factors

Exosome release by microglia, and their cargo content can be modulated by several inflammatory and neuronal-derived factors, and ultimately this modulation can shape the effect that microglial exosomes have on other cell types, such as neurons. For instance, a study on M9 microglial cell line primed with LPS shows exosomes from these cultures decreases neuronal viability of N2A by reducing the expression of syntaxin-1A (Tang et al., 2016). In addition, microglial cultures primed with LPS and Mn^2+^, show increased exosome release, containing higher levels of the inflammasome adaptor protein ASC (apoptosis-associated speck-like protein containing a caspase recruitment domain), which could then be incorporated by other microglial cultures, causing an increase of pro-IL-1β and NLRP3 expression levels (Sarkar et al., 2019). On the other hand, serum exosomes from welders, who are exposed to Mn^2+^, have higher levels of ASC and pro-inflammatory IL-17 and TNF-α; and when are added to microglia cultures, they increase the levels of NLRP3 and pro-IL-1β, indicating the ability of these exosomes to transfer ASC and induce inflammasome activation (Sarkar et al., 2019).

Also, exosomes from microglia and astrocytes activated with Th1 cytokines and LPS reduce the expression of proteins related to synapsis, such as synaptotagmin 1 (Syt1) and neuroligin 1 (Nlg1) in neuronal cultures, through the delivery of miR-146a-5p, which ultimately affects dendritic spine density (Prada et al., 2018). The authors of this work verified these results *in vivo*, by infusing exosomes derived from pro-inflammatory microglia, through osmotic mini pumps connected into the hippocampus, showing a decrease by 30% in the spine density (Prada et al., 2018). In a similar way, exosomes derived from BV2 cell culture submitted to stretch injury have reduced levels of miR-5121, which reduces the expression of synaptophysin, postsynaptic density protein 95 (PSD-95), and glutamate receptor 1 (GluR-1), decreasing dendritic spine density (Zhao et al., 2021).

BV2 cells incubated with an agonist of metabotropic glutamate receptor 5 (mGlu5), which exerts an anti-inflammatory effect on these cells, show increased exosome release, and when SH-SY5Y neurons are incubated with these exosomes, they become more susceptible to cell death induced by high doses of rotenone (Beneventano et al., 2017).

In ethanol stress models, ethanol administered to the diet of experimental rats increases exosomes release from the medial hypothalamus, while microglial cultures treated with ethanol also have higher numbers of exosomes. These exosomes are taken up by β-endorphin neurons in culture, inducing cell death (Mukherjee et al., 2020). Exosomes from microglia treated with ethanol showed augmented levels of aminopeptidase N (CD13), metalloproteinase-2 (MMP2) and the complement system protein C1q, the latter also detected in the hypothalamus of rats fed with ethanol and in neurons cultured with exosomes from ethanol-treated microglial cultures. Importantly, if microglia are inhibited *in vivo* with minocycline, C1q do not increase in the presence of ethanol, and cannot be detected in hypothalamic neurons, showing that microglial exosomes contribute to ethanol neurotoxicity through C1q activation and transference into neurons (Mukherjee et al., 2020).

In a high fat diet-induced inflammation *in vitro* model, exosomes derived from microglial cultures treated with sodium-palmitate, exhibit a proinflammatory profile that when added to hippocampal neuronal cultures, a significant reduction on dendritic spine maturation can be observed (Vinuesa et al., 2019).

On the other hand, in a rat model of focal cerebral ischemia, microglial cells increase their release of exosomes with higher levels of TNF-α and COX-2 in a glutaminase 1 (GLS-1) expression dependent manner (Gao et al., 2020), an enzyme previously associated with neuroinflammation. Pharmacological inhibition of GLS-1 reduced exosome release after focal cerebral ischemia and besides, it attenuates the infarction volume (Gao et al., 2020), showing exosome participation in the neuroinflammation spreading response.

Finally, exosomes from BV2 cultured under OGD exhibit higher expression of calcium/calmodulin-dependent 3′,5′-cyclic nucleotide phosphodiesterase 1B (PDE1-B) and induce higher neuronal cell death *in vivo*, when stereotaxically injected into the brain cortex of healthy mice. Pharmacological inhibition of PDE1-B can abrogate this effect. In addition, when BV2 exosomes under OGD are injected into the brain of mice with middle cerebral artery occlusion, they increase cell death, while PDE1-B inhibition protects against stroke-induced damage (Zang et al., 2020).

### Their Beneficial Effects

Protective effects of microglial derived exosomes have also been observed in neuronal cultures, through anti-inflammatory responses as well as modulation of synaptic activity and neurite growth (Table 2).

A study on primary hippocampal neurons cultured with micro vesicles (MVs) derived from primary microglia or N9 cell line, shows increased frequency and decay of miniature excitatory postsynaptic currents (mEPSC). Injection of microglial derived EV into the primary optical cortex of rats, increased evoked potentials *in vivo*, indicating enhanced excitatory synaptic activity (Antonucci et al., 2012). This effect was dependent on EV binding on neuronal membrane, and interestingly, broken EV by freeze and thaw cycles, depleted of their inner content, can recapitulate this effect in neuronal mEPSC, indicating that surface molecules present in EV are sufficient to stimulate exocytosis. In addition, the authors observed that EV promoted ceramide and sphingosine production in neuronal cultures, which were responsible for changes detected on synaptic activity (Antonucci et al., 2012).

Another study of how microglial derived exosomes can regulate synaptic activity shows that the presence of *N*-arachidonoylethanolamine (AEA) in the exosomes surface are able to stimulate type 1 cannabinoid receptor, inhibiting presynaptic transmission in GABAergic neurons (Gabrielli et al., 2015).

Exosomes from BV2 treated with brain extracts from repetitive mild traumatic brain injury (mTBI) are enriched in miR-124-3p (Huang et al., 2018), a miRNA abundant in the brain of mice subjected to mTBI (Li et al., 2019; Ge et al., 2020) that has been associated with anti-inflammatory M2 polarization of microglia (Yu et al., 2017; Huang et al., 2018). When mouse cortical neurons (Huang et al., 2018) and hippocampal neuronal cultures HT22 (Ge et al., 2020) are submitted to a scratch lesion and incubated with exosomes from BV2 over expressing miR-124-3p, they decrease their pro-inflammatory profile (Huang et al., 2018) and increase the expression of BDNF and neurogranin, augmenting neurites length and the number of branching (Huang et al., 2018; Ge et al., 2020). HT22 cultures after scratch increase the expression of autophagy and apoptosis related proteins, and this is attenuated when co-cultured with BV2 overexpressing miR-124-3p, only when exosome release is allowed (Li et al., 2019). They also decrease the expression of Rela (a target of miR-124-3p, strongly associated with neuroinflammation and neurodegeneration), intracellular calcium sensor VILIP-1 (also a neurodegenerative marker), Aβ and APP protein *in vitro* and, *in vivo*, when they are intravenously administrated in mice after 35 days of brain lesion (Ge et al., 2020). In addition, administration of miR-124-3p enriched microglial exosomes improve both motor and cognitive performance, indicating a protective effect in this neuronal injury model (Huang et al., 2018; Li et al., 2019; Ge et al., 2020). Also, in a mice model of ischemia, miR-124 enriched exosomes have shown protective effects. Exosomes from BV2 polarized into anti-inflammatory M2 phenotype, by IL-4, have higher levels of miR-124 (Song et al., 2019) and miR-137 (Zhang et al., 2021) and taken up by primary neuronal cultures, protecting them against OGD-induced cell death *in vitro* and *in vivo*. Moreover, intravenous administration of these exosomes increases neuronal survival and reduces the infarct volume, after 3 days. The authors showed that the neuroprotective effect was dependent on miR-124, so when its expression is inhibited, the favorable outcome is eliminated (Song et al., 2019).

An interesting study on glioblastoma, where the authors make a distinction between MVs and exosomes, shows that only MVs secreted by BV2 or mice primary microglia can suppress migration and invasion capacity of glioblastoma cell line GL261, when microglia are treated with LPS and interferon (IFN)-γ (Grimaldi et al., 2019). The same MVs can also decrease tumor cells viability in the presence of neuronal cultures, and they reduce tumor size in mice injected with GL261 cells, when MVs are infused through a brain cannula. Analysis of brain slices showed incorporation of MVs into microglia present in the tumor, which had reduced expression of anti-inflammatory, and thereby anti-tumor genes, such as arg1, CD206 and CD163 (Grimaldi et al., 2019).

## Oligodendrocyte and Schwan Cells-Derived Exosomes

It has been observed that exosome secretion from oligodendrocytes can be stimulated by neuronal signals, such as glutamate and other AMPA and NMDA agonist, in a Ca^2+^ entry dependent manner, as shown by direct incubation of oligodendrocytes with glutamate, or co-culturing with primary cortical neurons stimulated with potassium in a Boyden chamber system (Fruhbeis et al., 2013). Using the same co-culturing method, the authors also show that exosomes from oligodendrocytes are mainly internalized by microglia, and in a lesser extent by neurons (Fruhbeis et al., 2013). Other authors, however, have shown that exosomes from oligodendrocytes cell line OliNeu are practically just incorporated by microglia *in vitro* and *in vivo*, mainly by micropinocytosis (Fitzner et al., 2011). Addition of these exosomes to microglial cultures did not induce pro-inflammatory cytokine release under basal conditions, nor affected the release of pro-inflammatory molecules when stimulated with LPS *in vitro*, indicating they do not activate microglia (Fitzner et al., 2011).

In neuronal cultures, exosomes from oligodendrocytes exert protective effect against H_2_O_2_-induced oxidative stress and nutrient deprivation stress (Fruhbeis et al., 2013) (Table 3). When neurons are co-cultured with oligodendrocytes in a Boyden chamber system and subjected to an *in vitro* stroke model of OGD they exhibit a higher metabolic rate, and are protected from oxidative stress, due to exosome transfer of SOD and catalase, from oligodendrocytes (Frohlich et al., 2014). In addition, using multi-electrode array measurements of basal neuronal activity in primary cortical neurons, has shown that exosomes from oligodendrocyte increase neuronal action potential fire rate (Frohlich et al., 2014).

**TABLE 3:** Summary of neuroprotective and neurotoxic effects of oligodendrocytes and Schwann cells-derived exosomes with identified cargo.

Role	Stimuli/disease	Cargo	Effect observed	References
Neurotoxic	PLP and CNP deficiency	Decreased PLP levels	Reduces axonal transport under nutrient deprivation	Frühbeis et al., 2020
	Polarization into repair phenotype with forskolin	MiR-21	Increases axonal regeneration	López-Leal and Díaz-Viraqué, 2020
Neuroprotective	OGD	SOD and catalase	Increases metabolic rate and neuroprotection under oxidative stress	Frohlich et al., 2014
	–	Myelin antigen	Decreases neuroinflammation, demyelination and axonal damage	Casella et al., 2020
Neuroprotective in a EAE model	IL-4	miR-124	Protects neurons from OGD induced cell death	Song et al., 2019
Regulatory	–	PLP, CNP	Inhibits myelination of neurons	Krämer-Albers et al., 2007

Another study has shown that exposure of primary hippocampal neurons to exosomes from oligodendrocytes reduces their vesicle pauses during axonal movement, under normal conditions, and restore vesicle mobility under oxidative stress and nutrient deprivation, by increasing anterograde and retrograde movement along the axons (Frühbeis et al., 2020).

Interestingly, exosomes from oligodendrocytes, which are enriched in myelin tetraspan protein (PLP) and 2′,3′-cyclic nucleotide 3′-phosphodiesterase (CNP) (Krämer-Albers et al., 2007), inhibit myelination of primary neurons co-cultured with oligodendrocytes, due to their enrichment in PLP. However, if oligodendrocytes are incubated with neuronal conditioned media, PLP levels on exosomes are reduced, suggesting oligodendrocytes-exosomes might participate in feedback mechanism regulating myelin synthesis (Bakhti et al., 2011).

Mice models deficient for PLP or CNP suffer from secondary axonal degeneration and exhibit intracellular accumulation of multivesicular bodies, *in vivo*, with decreased reduction in exosome release capacity *in vitro*. Moreover, PLP and CNP deficiency decreases the level of cargo transfer of oligodendrocytes exosomes and loses the ability to sustain axonal transport in neurons under nutrient deprivation conditions, even when the number of exosomes are normalized with control counterparts (Frühbeis et al., 2020). Altogether, these data suggest that although exosomes from oligodendrocytes might be predominately captured by microglia, they have protective effects on neurons that are highly dependent on PLP and CNP expression in oligodendrocytes.

Interestingly, a study shows that IV administration of exosomes from mature oligodendrocytes, which are enriched in myelin antigens, improve the clinical score and increases survival rate in mice models of experimental autoimmune encephalitis (EAE). These exosomes reduced CD4+ T cell infiltration into the CNS and decreased demyelination and axonal damage (Casella et al., 2020).

Lastly, regarding Schwan cell derived-exosomes, studies have shown that they are internalized by dorsal root ganglia (DRG) neurons when co-cultured with Schwan cells in Boyden chambers, and that they stimulate axonal growth in DGR explants, promoting axonal regeneration and growth cone extension after mechanical injury *in vitro* (Lopez-Verrilli et al., 2013). When Schwann cells exosomes were injected into crushed sciatic nerves, they were also internalized by neurons, specifically in their axons, increasing nerve regeneration after 4 days (Lopez-Verrilli et al., 2013). This pro-regenerative capacity of Schwann cells-derived exosomes was observed only to occur if the cells were polarized into a repair phenotype, and it depended on the miR-21 presence in exosomes (López-Leal and Díaz-Viraqué, 2020).

In addition, exosomes from human Schwann cell line RSC9 cultures increases proliferation and decreases apoptosis in DRG cultures submitted to mechanical strain (Zhou et al., 2018).

## Conclusion and Future Directions

We have summarized the most recent evidence regarding glial derived-exosome detrimental or beneficial effects upon neurons and other brain cells, in the context of different pathological settings. Inflammatory conditions found in most neurological diseases can alter the cargo content of exosomes, and therefore their neuroprotective properties. For this reason, it is necessary to refine or develop better imaging strategies that allow tracking of cell-specific exosome secretion and internalization. The latter, in combination with exosome labeling and capture techniques, coupled with transcriptomics, metabolomics and proteomics approaches would deeply increase our understanding on how EVs cargo are modulated when submitted to cell microenvironment variations *in vivo*, as this can also affect experimental interpretations and therapeutic applications of exosomes.

In this regard, *in vitro* and *ex vivo* studies have allowed the identification of key proteins and miRNAs that are enriched in glial exosomes can promote neuronal viability, modulate their synaptic transmission and support axonal growth (Table 1), however, significant amount of literature does not analyze the nature of exosome content, and therefore, the root of the effects described either.

Furthermore, most of the data available on glial-exosomes, their cargo and their biological effect come from studies where exosomes are isolated from cell culture supernatants, posing the need of more studies that use methodological approaches to specifically isolate glial-derived exosomes from body fluids of healthy people and patients suffering from neurological and neuropsychiatric disorders.

Some studies already suggest that finding higher levels of glial-derived exosomes in blood could be used as a disease biomarker in TBI (Manek et al., 2018; Mondello et al., 2020; Wang et al., 2021), EAE (Willis et al., 2017), and alcohol abuse (Kodidela et al., 2020). Therefore, if new studies arise in the field, connecting number, glial origin and cargo, their assessment would be expected to be useful as a real biomarker for disease.

The authors of this review consider that in the near future, exosome analysis from body fluids will provide a more comprehensive and accurate assessment of neurological disease development, but also, that the information about exosomes cargo will translate into synthetic exosome production that could provide a safer and robust line of treatment.

## Author Contributions

KO and RE-V wrote the manuscript with the input of MC, RL, CG, AV-L, MG-R, and PL-C. All authors contributed to the article and approved the submitted version.

## Conflict of Interest

The authors declare that the research was conducted in the absence of any commercial or financial relationships that could be construed as a potential conflict of interest.

## Publisher’s Note

All claims expressed in this article are solely those of the authors and do not necessarily represent those of their affiliated organizations, or those of the publisher, the editors and the reviewers. Any product that may be evaluated in this article, or claim that may be made by its manufacturer, is not guaranteed or endorsed by the publisher.

## References

[B1] Antonucci F. Turola E. Riganti L. Caleo M. Gabrielli M. Perrotta C. (2012). Microvesicles released from microglia stimulate synaptic activity via enhanced sphingolipid metabolism. *EMBO J.* 31 1231–1240. 10.1038/emboj.2011.489 22246184 PMC3297996

[B2] Asai H. Ikezu S. Tsunoda S. Medalla M. Luebke J. Haydar T. (2015). Depletion of microglia and inhibition of exosome synthesis halt tau propagation. *Nat. Neurosci.* 18 1584–1593. 10.1038/nn.4132 26436904 PMC4694577

[B3] Bakhti M. Winter C. Simons M. (2011). Inhibition of myelin membrane sheath formation by oligodendrocyte-derived exosome-like vesicles. *J. Biol. Chem.* 286 787–796. 10.1074/jbc.M110.190009 20978131 PMC3013037

[B4] Beere H. M. Green D. R. (2001). Stress management - heat shock protein-70 and the regulation of apoptosis. *Trends Cell Biol.* 11 6–10. 10.1016/s0962-8924(00)01874-2 11146277

[B5] Beneventano M. Spampinato S. F. Merlo S. Chisari M. Platania P. Ragusa M. (2017). Shedding of microvesicles from microglia contributes to the effects induced by metabotropic glutamate receptor 5 activation on neuronal death. *Front. Pharmacol.* 8:812. 10.3389/fphar.2017.00812 29170640 PMC5684115

[B6] Beretta C. Nikitidou E. Streubel-Gallasch L. Ingelsson M. Sehlin D. Erlandsson A. (2020). Extracellular vesicles from amyloid-beta exposed cell cultures induce severe dysfunction in cortical neurons. *Sci. Rep.* 10:19656. 10.1038/s41598-020-72355-2 33184307 PMC7661699

[B7] Blanco A. M. Valles S. L. Pascual M. Guerri C. (2005). Involvement of TLR4/type I IL-1 receptor signaling in the induction of inflammatory mediators and cell death induced by ethanol in cultured astrocytes. *J. Immunol.* 175 6893–6899. 10.4049/jimmunol.175.10.6893 16272348

[B8] Bu X. Li D. Wang F. Sun Q. Zhang Z. (2020). Protective role of astrocyte-derived exosomal microRNA-361 in cerebral ischemic-reperfusion injury by regulating the AMPK/mTOR Signaling Pathway and Targeting CTSB. *Neuropsychiatr. Dis. Treat.* 16 1863–1877. 10.2147/NDT.S260748 32801720 PMC7410492

[B9] Casella G. Rasouli J. Boehm A. Zhang W. Xiao D. Ishikawa L. L. W. (2020). Oligodendrocyte-derived extracellular vesicles as antigen-specific therapy for autoimmune neuroinflammation in mice. *Sci. Transl. Med.* 12:eaba0599. 10.1126/scitranslmed.aba0599 33148622 PMC7886371

[B10] Chang C. Lang H. Geng N. Wang J. Li N. Wang X. (2013). Exosomes of BV-2 cells induced by alpha-synuclein: important mediator of neurodegeneration in PD. *Neurosci. Lett.* 548 190–195. 10.1016/j.neulet.2013.06.009 23792198

[B11] Chaudhuri A. D. Dastgheyb R. M. Yoo S. W. Trout A. Talbot C. C. Jr. Hao H. (2018). TNFalpha and IL-1beta modify the miRNA cargo of astrocyte shed extracellular vesicles to regulate neurotrophic signaling in neurons. *Cell Death Dis.* 9:363. 10.1038/s41419-018-0369-4 29507357 PMC5838212

[B12] Chen W. Zheng P. Hong T. Wang Y. Liu N. He B. (2020). Astrocytes-derived exosomes induce neuronal recovery after traumatic brain injury via delivering gap junction alpha 1-20 k. *J. Tissue Eng. Regen. Med.* 14 412–423. 10.1002/term.3002 31826322

[B13] Chun C. Smith A. S. T. Kim H. Kamenz D. S. Lee J. H. Lee J. B. (2021). Astrocyte-derived extracellular vesicles enhance the survival and electrophysiological function of human cortical neurons *in vitro*. *Biomaterials* 271:120700. 10.1016/j.biomaterials.2021.120700 33631652 PMC8044026

[B14] Clayton K. Delpech J. C. Herron S. Iwahara N. Ericsson M. Saito T. (2021). Plaque associated microglia hyper-secrete extracellular vesicles and accelerate tau propagation in a humanized APP mouse model. *Mol. Neurodegener.* 16:18. 10.1186/s13024-021-00440-9 PMC7986521 33752701

[B15] Cohn W. Melnik M. Huang C. Teter B. Chandra S. Zhu C. (2021). Multi-omics analysis of microglial extracellular vesicles from human Alzheimer’s disease brain tissue reveals disease-associated signatures. *Front. Pharmacol.* 12:766082. 10.3389/fphar.2021.766082 34925024 PMC8675946

[B16] Dassati S. Waldner A. Schweigreiter R. (2014). Apolipoprotein D takes center stage in the stress response of the aging and degenerative brain. *Neurobiol. Aging* 35 1632–1642. 10.1016/j.neurobiolaging.2014.01.148 24612673 PMC3988949

[B17] Dinkins M. B. Dasgupta S. Wang G. Zhu G. Bieberich E. (2014). Exosome reduction in vivo is associated with lower amyloid plaque load in the 5XFAD mouse model of Alzheimer’s disease. *Neurobiol. Aging* 35 1792–1800. 10.1016/j.neurobiolaging.2014.02.012 24650793 PMC4035236

[B18] Du L. Jiang Y. Sun Y. (2021). Astrocyte-derived exosomes carry microRNA-17-5p to protect neonatal rats from hypoxic-ischemic brain damage via inhibiting BNIP-2 expression. *Neurotoxicology* 83 28–39. 10.1016/j.neuro.2020.12.006 33309839

[B19] Fernandez-Lizarbe S. Pascual M. Guerri C. (2009). Critical role of TLR4 response in the activation of microglia induced by ethanol. *J. Immunol.* 183 4733–4744. 10.4049/jimmunol.0803590 19752239

[B20] Fitzner D. Schnaars M. Van Rossum D. Krishnamoorthy G. Dibaj P. Bakhti M. (2011). Selective transfer of exosomes from oligodendrocytes to microglia by macropinocytosis. *J. Cell Sci.* 124 447–458. 10.1242/jcs.074088 21242314

[B21] Froger N. Matonti F. Roubeix C. Forster V. Ivkovic I. Brunel N. (2020). VEGF is an autocrine/paracrine neuroprotective factor for injured retinal ganglion neurons. *Sci. Rep.* 10:12409. 10.1038/s41598-020-68488-z 32710087 PMC7382485

[B22] Frohlich D. Kuo W. P. Fruhbeis C. Sun J. J. Zehendner C. M. Luhmann H. J. (2014). Multifaceted effects of oligodendroglial exosomes on neurons: impact on neuronal firing rate, signal transduction and gene regulation. *Philos. Trans. R. Soc. Lond. B Biol. Sci.* 369:20130510. 10.1098/rstb.2013.0510 25135971 PMC4142031

[B23] Fruhbeis C. Frohlich D. Kuo W. P. Amphornrat J. Thilemann S. Saab A. S. (2013). Neurotransmitter-triggered transfer of exosomes mediates oligodendrocyte-neuron communication. *PLoS Biol.* 11:e1001604. 10.1371/journal.pbio.1001604 23874151 PMC3706306

[B24] Frühbeis C. Kuo-Elsner W. P. Muller C. Barth K. Peris L. Tenzer S. (2020). Oligodendrocytes support axonal transport and maintenance via exosome secretion. *PLoS Biol.* 18:e3000621. 10.1371/journal.pbio.3000621 33351792 PMC7787684

[B25] Gabrielli M. Battista N. Riganti L. Prada I. Antonucci F. Cantone L. (2015). Active endocannabinoids are secreted on extracellular membrane vesicles. *EMBO Rep.* 16 213–220. 10.15252/embr.201439668 25568329 PMC4328748

[B26] Gao G. Li C. Zhu J. Wang Y. Huang Y. Zhao S. (2020). Glutaminase 1 regulates neuroinflammation after cerebral ischemia through enhancing microglial activation and pro-inflammatory exosome release. *Front. Immunol.* 11:161. 10.3389/fimmu.2020.00161 32117296 PMC7020613

[B27] Ge X. Guo M. Hu T. Li W. Huang S. Yin Z. (2020). Increased Microglial Exosomal miR-124-3p Alleviates Neurodegeneration and Improves Cognitive Outcome after rmTBI. *Mol. Ther.* 28 503–522. 10.1016/j.ymthe.2019.11.017 31843449 PMC7001001

[B28] Goetzl E. J. Mustapic M. Kapogiannis D. Eitan E. Lobach I. V. Goetzl L. (2016). Cargo proteins of plasma astrocyte-derived exosomes in Alzheimer’s disease. *FASEB J.* 30 3853–3859. 10.1096/fj.201600756R 27511944 PMC5067254

[B29] Goetzl E. J. Schwartz J. B. Abner E. L. Jicha G. A. Kapogiannis D. (2018). High complement levels in astrocyte-derived exosomes of Alzheimer disease. *Ann. Neurol.* 83 544–552. 10.1002/ana.25172 29406582 PMC5867263

[B30] Gosselin R. D. Meylan P. Decosterd I. (2013). Extracellular microvesicles from astrocytes contain functional glutamate transporters: regulation by protein kinase C and cell activation. *Front. Cell. Neurosci.* 7:251. 10.3389/fncel.2013.00251 24368897 PMC3857901

[B31] Grimaldi A. Serpe C. Chece G. Nigro V. Sarra A. Ruzicka B. (2019). Microglia-derived microvesicles affect microglia phenotype in glioma. *Front. Cell. Neurosci.* 13:41. 10.3389/fncel.2019.00041 30853898 PMC6395438

[B32] Guitart K. Loers G. Buck F. Bork U. Schachner M. Kleene R. (2016). Improvement of neuronal cell survival by astrocyte-derived exosomes under hypoxic and ischemic conditions depends on prion protein. *Glia* 64 896–910. 10.1002/glia.22963 26992135

[B33] Guo M. Wang J. Zhao Y. Feng Y. Han S. Dong Q. (2020). Microglial exosomes facilitate α-synuclein transmission in Parkinson’s disease. *Brain* 143 1476–1497. 10.1093/brain/awaa090 32355963 PMC7241957

[B34] Hajj G. N. Arantes C. P. Dias M. V. Roffe M. Costa-Silva B. Lopes M. H. (2013). The unconventional secretion of stress-inducible protein 1 by a heterogeneous population of extracellular vesicles. *Cell. Mol. Life Sci.* 70 3211–3227. 10.1007/s00018-013-1328-y 23543276 PMC11113396

[B35] Huang S. Ge X. Yu J. Han Z. Yin Z. Li Y. (2018). Increased miR-124-3p in microglial exosomes following traumatic brain injury inhibits neuronal inflammation and contributes to neurite outgrowth via their transfer into neurons. *FASEB J.* 32 512–528. 10.1096/fj.201700673R 28935818

[B36] Ibanez F. Montesinos J. Urena-Peralta J. R. Guerri C. Pascual M. (2019). TLR4 participates in the transmission of ethanol-induced neuroinflammation via astrocyte-derived extracellular vesicles. *J. Neuroinflammation* 16:136. 10.1186/s12974-019-1529-x 31272469 PMC6610989

[B37] Iovino L. Tremblay M. E. Civiero L. (2020). Glutamate-induced excitotoxicity in Parkinson’s disease: the role of glial cells. *J. Pharmacol. Sci.* 144 151–164. 10.1016/j.jphs.2020.07.011 32807662

[B38] Jin T. Gu J. Li Z. Xu Z. Gui Y. (2021). Recent advances on extracellular vesicles in central nervous system diseases. *Clin. Interv. Aging* 16 257–274. 10.2147/CIA.S288415 33603351 PMC7882422

[B39] Joshi P. Turola E. Ruiz A. Bergami A. Libera D. D. Benussi L. (2014). Microglia convert aggregated amyloid-beta into neurotoxic forms through the shedding of microvesicles. *Cell Death Differ.* 21 582–593. 10.1038/cdd.2013.180 24336048 PMC3950321

[B40] Kodidela S. Gerth K. Sinha N. Kumar A. Kumar P. Kumar S. (2020). Circulatory Astrocyte and Neuronal EVs as Potential Biomarkers of Neurological Dysfunction in HIV-Infected Subjects and Alcohol/Tobacco Users. *Diagnostics* 10:349. 10.3390/diagnostics10060349 32481515 PMC7345258

[B41] Krämer-Albers E. M. Bretz N. Tenzer S. Winterstein C. Mobius W. Berger H. (2007). Oligodendrocytes secrete exosomes containing major myelin and stress-protective proteins: Trophic support for axons? *Proteomics Clin. Appl.* 1 1446–1461. 10.1002/prca.200700522 21136642

[B42] Kumar A. Kim S. Su Y. Sharma M. Kumar P. Singh S. (2021). Brain cell-derived exosomes in plasma serve as neurodegeneration biomarkers in male cynomolgus monkeys self-administrating oxycodone. *EBioMedicine* 63:103192. 10.1016/j.ebiom.2020.103192 33418508 PMC7804975

[B43] Lange C. Storkebaum E. De Almodovar C. R. Dewerchin M. Carmeliet P. (2016). Vascular endothelial growth factor: a neurovascular target in neurological diseases. *Nat. Rev. Neurol.* 12 439–454. 10.1038/nrneurol.2016.88 27364743

[B44] Li D. Huang S. Yin Z. Zhu J. Ge X. Han Z. (2019). Increases in miR-124-3p in microglial exosomes confer neuroprotective effects by targeting FIP200-mediated neuronal autophagy following traumatic brain injury. *Neurochem. Res.* 44 1903–1923. 10.1007/s11064-019-02825-1 31190315

[B45] Li H. Ruberu K. Munoz S. S. Jenner A. M. Spiro A. Zhao H. (2015). Apolipoprotein D modulates amyloid pathology in APP/PS1 Alzheimer’s disease mice. *Neurobiol. Aging* 36 1820–1833. 10.1016/j.neurobiolaging.2015.02.010 25784209

[B46] Li Z. Moniruzzaman M. Dastgheyb R. M. Yoo S. W. Wang M. Hao H. (2020). Astrocytes deliver CK1 to neurons via extracellular vesicles in response to inflammation promoting the translation and amyloidogenic processing of APP. *J. Extracell. Vesicles* 10 e12035. 10.1002/jev2.12035 33408815 PMC7775567

[B47] Liu W. Bai X. Zhang A. Huang J. Xu S. Zhang J. (2019). Role of exosomes in central nervous system diseases. *Front. Mol. Neurosci.* 12:240. 10.3389/fnmol.2019.00240 31636538 PMC6787718

[B48] Long X. Yao X. Jiang Q. Yang Y. He X. Tian W. (2020). Astrocyte-derived exosomes enriched with miR-873a-5p inhibit neuroinflammation via microglia phenotype modulation after traumatic brain injury. *J. Neuroinflammation* 17:89. 10.1186/s12974-020-01761-0 32192523 PMC7082961

[B49] López-Leal R. Díaz-Viraqué F. (2020). Schwann cell reprogramming into repair cells increases miRNA-21 expression in exosomes promoting axonal growth. *J. Cell Sci.* 133:jcs239004. 10.1242/jcs.239004 32409566

[B50] Lopez-Verrilli M. A. Picou F. Court F. A. (2013). Schwann cell-derived exosomes enhance axonal regeneration in the peripheral nervous system. *Glia* 61 1795–1806. 10.1002/glia.22558 24038411

[B51] Luarte A. Henzi R. Fernandez A. Gaete D. Cisternas P. Pizarro M. (2020). Astrocyte-derived small extracellular vesicles regulate dendritic complexity through miR-26a-5p activity. *Cells* 9:930. 10.3390/cells9040930 32290095 PMC7226994

[B52] Manek R. Moghieb A. Yang Z. Kumar D. Kobessiy F. Sarkis G. A. (2018). Protein biomarkers and neuroproteomics characterization of microvesicles/exosomes from human cerebrospinal fluid following traumatic brain injury. *Mol. Neurobiol.* 55 6112–6128. 29188495 10.1007/s12035-017-0821-y PMC6359938

[B53] Mao S. Sun Q. Xiao H. Zhang C. Li L. (2015). Secreted miR-34a in astrocytic shedding vesicles enhanced the vulnerability of dopaminergic neurons to neurotoxins by targeting Bcl-2. *Protein Cell* 6 529–540. 10.1007/s13238-015-0168-y 26091620 PMC4491052

[B54] Marote A. Teixeira F. G. Mendes-Pinheiro B. Salgado A. J. (2016). MSCs-derived exosomes: cell-secreted nanovesicles with regenerative potential. *Front. Pharmacol.* 7:231. 10.3389/fphar.2016.00231 27536241 PMC4971062

[B55] Mathieu M. Martin-Jaular L. Lavieu G. Thery C. (2019). Specificities of secretion and uptake of exosomes and other extracellular vesicles for cell-to-cell communication. *Nat. Cell Biol.* 21 9–17. 10.1038/s41556-018-0250-9 30602770

[B56] Matsuzaki H. Tamatani M. Yamaguchi A. Namikawa K. Kiyama H. Vitek M. P. (2001). Vascular endothelial growth factor rescues hippocampal neurons from glutamate-induced toxicity: signal transduction cascades. *FASEB J.* 15 1218–1220. 11344093

[B57] Mondello S. Guedes V. A. Lai C. Czeiter E. Amrein K. Kobeissy F. (2020). Circulating brain injury exosomal proteins following moderate-to-severe traumatic brain injury: temporal profile, outcome prediction and therapy implications. *Cells* 9:977. 10.3390/cells9040977 32326450 PMC7227241

[B58] Mukherjee S. Cabrera M. A. Boyadjieva N. I. Berger G. Rousseau B. Sarkar D. K. (2020). Alcohol increases exosome release from microglia to promote complement C1q-induced cellular death of proopiomelanocortin neurons in the hypothalamus in a rat model of fetal alcohol spectrum disorders. *J. Neurosci.* 40 7965–7979. 10.1523/JNEUROSCI.0284-20.2020 32887744 PMC7548688

[B59] Navarro A. Rioseras B. Del Valle E. Martinez-Pinilla E. Astudillo A. Tolivia J. (2018). Expression pattern of myelin-related apolipoprotein D in human multiple sclerosis lesions. *Front. Aging Neurosci.* 10:254. 10.3389/fnagi.2018.00254 30186153 PMC6110904

[B60] Novoselova T. V. Margulis B. A. Novoselov S. S. Sapozhnikov A. M. Van Der Spuy J. Cheetham M. E. (2005). Treatment with extracellular HSP70/HSC70 protein can reduce polyglutamine toxicity and aggregation. *J. Neurochem.* 94 597–606. 10.1111/j.1471-4159.2005.03119.x 15992387

[B61] Ohmichi T. Mitsuhashi M. Tatebe H. Kasai T. Ali El-Agnaf O. M. Tokuda T. (2019). Quantification of brain-derived extracellular vesicles in plasma as a biomarker to diagnose Parkinson’s and related diseases. *Parkinsonism Relat. Disord.* 61 82–87. 10.1016/j.parkreldis.2018.11.021 30502924

[B62] Onodera T. Sakudo A. Tsubone H. Itohara S. (2014). Review of studies that have used knockout mice to assess normal function of prion protein under immunological or pathophysiological stress. *Microbiol. Immunol.* 58 361–374. 10.1111/1348-0421.12162 24866463

[B63] Pascua-Maestro R. Gonzalez E. Lillo C. Ganfornina M. D. Falcon-Perez J. M. Sanchez D. (2019). Extracellular vesicles secreted by astroglial cells transport apolipoprotein D to neurons and mediate neuronal survival upon oxidative stress. *Front. Cell. Neurosci.* 12:526. 10.3389/fncel.2018.00526 30687015 PMC6335244

[B64] Patel M. R. Weaver A. M. (2021). Astrocyte-derived small extracellular vesicles promote synapse formation via fibulin-2-mediated TGF-beta signaling. *Cell Rep.* 34:108829. 10.1016/j.celrep.2021.108829 33691102 PMC8002899

[B65] Pei X. Li Y. Zhu L. Zhou Z. (2020). Astrocyte-derived exosomes transfer miR-190b to inhibit oxygen and glucose deprivation-induced autophagy and neuronal apoptosis. *Cell Cycle* 19 906–917. 10.1080/15384101.2020.1731649 32150490 PMC7217362

[B66] Prada I. Gabrielli M. Turola E. Iorio A. D’Arrigo G. Parolisi R. (2018). Glia-to-neuron transfer of miRNAs via extracellular vesicles: a new mechanism underlying inflammation-induced synaptic alterations. *Acta Neuropathol.* 135 529–550. 10.1007/s00401-017-1803-x 29302779 PMC5978931

[B67] Proia P. Schiera G. Mineo M. Ingrassia A. M. Santoro G. Savettieri G. (2008). Astrocytes shed extracellular vesicles that contain fibroblast growth factor-2 and vascular endothelial growth factor. *Int. J. Mol. Med.* 21 63–67. 18097617

[B68] Rastogi S. Sharma V. Bharti P. S. Rani K. Modi G. P. Nikolajeff F. (2021). The evolving landscape of exosomes in neurodegenerative diseases: exosomes characteristics and a promising role in early diagnosis. *Int. J. Mol. Sci.* 22:440. 10.3390/ijms22010440 33406804 PMC7795439

[B69] Sarkar S. Rokad D. Malovic E. Luo J. Harischandra D. S. Jin H. (2019). Manganese activates NLRP3 inflammasome signaling and propagates exosomal release of ASC in microglial cells. *Sci. Signal.* 12:eaat9900. 10.1126/scisignal.aat9900 30622196 PMC6420319

[B70] Shaimardanova A. A. Solovyeva V. V. Chulpanova D. S. James V. Kitaeva K. V. Rizvanov A. A. (2020). Extracellular vesicles in the diagnosis and treatment of central nervous system diseases. *Neural Regen. Res.* 15 586–596. 10.4103/1673-5374.266908 31638080 PMC6975137

[B71] Shim J. W. Madsen J. R. (2018). VEGF Signaling in Neurological Disorders. *Int. J. Mol. Sci.* 19:275. 10.3390/ijms19010275 29342116 PMC5796221

[B72] Silverman J. M. Christy D. Shyu C. C. Moon K. M. Fernando S. Gidden Z. (2019). CNS-derived extracellular vesicles from superoxide dismutase 1 (SOD1)(G93A) ALS mice originate from astrocytes and neurons and carry misfolded SOD1. *J. Biol. Chem.* 294 3744–3759. 10.1074/jbc.RA118.004825 30635404 PMC6416428

[B73] Song Y. Li Z. He T. Qu M. Jiang L. Li W. (2019). M2 microglia-derived exosomes protect the mouse brain from ischemia-reperfusion injury via exosomal miR-124. *Theranostics* 9 2910–2923. 10.7150/thno.30879 31244932 PMC6568171

[B74] Svensson B. Peters M. Konig H. G. Poppe M. Levkau B. Rothermundt M. (2002). Vascular endothelial growth factor protects cultured rat hippocampal neurons against hypoxic injury via an antiexcitotoxic, caspase-independent mechanism. *J. Cereb. Blood Flow Metab.* 22 1170–1175. 10.1097/01.wcb.0000037988.07114.98 12368654

[B75] Tang L. L. Wu Y. B. Fang C. Q. Qu P. Gao Z. L. (2016). NDRG2 promoted secreted miR-375 in microvesicles shed from M1 microglia, which induced neuron damage. *Biochem. Biophys. Res. Commun.* 469 392–398. 10.1016/j.bbrc.2015.11.098 26631961

[B76] Taylor A. R. Robinson M. B. Gifondorwa D. J. Tytell M. Milligan C. E. (2007). Regulation of heat shock protein 70 release in astrocytes: role of signaling kinases. *Dev. Neurobiol.* 67 1815–1829. 10.1002/dneu.20559 17701989

[B77] Tidwell J. L. Houenou L. J. Tytell M. (2004). Administration of Hsp70 *in vivo* inhibits motor and sensory neuron degeneration. *Cell Stress Chaperones* 9 88–98. 15270081 10.1379/CSC-9R.1 PMC1065310

[B78] Todd A. C. Hardingham G. E. (2020). The regulation of astrocytic glutamate transporters in health and neurodegenerative diseases. *Int. J. Mol. Sci.* 21:9607. 10.3390/ijms21249607 33348528 PMC7766851

[B79] Upadhya R. Zingg W. Shetty S. Shetty A. K. (2020). Astrocyte-derived extracellular vesicles: neuroreparative properties and role in the pathogenesis of neurodegenerative disorders. *J. Control. Release* 323 225–239. 10.1016/j.jconrel.2020.04.017 32289328 PMC7299747

[B80] Vabulas R. M. Ahmad-Nejad P. Ghose S. Kirschning C. J. Issels R. D. Wagner H. (2002). HSP70 as endogenous stimulus of the Toll/interleukin-1 receptor signal pathway. *J. Biol. Chem.* 277 15107–15112. 10.1074/jbc.M111204200 11842086

[B81] Varcianna A. Myszczynska M. A. Castelli L. M. O’neill B. Kim Y. Talbot J. (2019). Micro-RNAs secreted through astrocyte-derived extracellular vesicles cause neuronal network degeneration in C9orf72 ALS. *EBioMedicine* 40 626–635. 10.1016/j.ebiom.2018.11.067 30711519 PMC6413467

[B82] Venturini A. Passalacqua M. Pelassa S. Pastorino F. Tedesco M. Cortese K. (2019). Exosomes from astrocyte processes: signaling to neurons. *Front. Pharmacol.* 10:1452. 10.3389/fphar.2019.01452 31849688 PMC6901013

[B83] Vinuesa A. Bentivegna M. Calfa G. Filipello F. Pomilio C. Bonaventura M. M. (2019). Early Exposure to a High-Fat Diet Impacts on Hippocampal Plasticity: Implication of Microglia-Derived Exosome-like Extracellular Vesicles. *Mol Neurobiol* 56 5075–5094. 10.1007/s12035-018-1435-8 30474797

[B84] Wang G. Dinkins M. He Q. Zhu G. Poirier C. Campbell A. (2012). Astrocytes secrete exosomes enriched with proapoptotic ceramide and prostate apoptosis response 4 (PAR-4): potential mechanism of apoptosis induction in Alzheimer disease (AD). *J. Biol. Chem.* 287 21384–21395. 10.1074/jbc.M112.340513 22532571 PMC3375560

[B85] Wang S. Cesca F. Loers G. Schweizer M. Buck F. Benfenati F. (2011). Synapsin I is an oligomannose-carrying glycoprotein, acts as an oligomannose-binding lectin, and promotes neurite outgrowth and neuronal survival when released via glia-derived exosomes. *J. Neurosci.* 31 7275–7290. 10.1523/JNEUROSCI.6476-10.2011 21593312 PMC6622588

[B86] Wang Z. Wang H. Becker R. Rufo J. Yang S. Mace B. E. (2021). Acoustofluidic separation enables early diagnosis of traumatic brain injury based on circulating exosomes. *Microsyst. Nanoeng.* 7:20. 10.1038/s41378-021-00244-3 34567734 PMC8433131

[B87] Willis C. M. Menoret A. Jellison E. R. Nicaise A. M. Vella A. T. Crocker S. J. (2017). A Refined Bead-Free Method to Identify Astrocytic Exosomes in Primary Glial Cultures and Blood Plasma. *Front Neurosci* 11:335. 10.3389/fnins.2017.00335 28663721 PMC5471332

[B88] Winston C. N. Romero H. K. Ellisman M. Nauss S. Julovich D. A. Conger T. (2019). Assessing neuronal and astrocyte derived exosomes from individuals with mild traumatic brain injury for markers of neurodegeneration and cytotoxic activity. *Front. Neurosci.* 13:1005. 10.3389/fnins.2019.01005 31680797 PMC6797846

[B89] Xia Y. Zhang G. Han C. Ma K. Guo X. Wan F. (2019). Microglia as modulators of exosomal alpha-synuclein transmission. *Cell Death Dis.* 10:174. 10.1038/s41419-019-1404-9 30787269 PMC6382842

[B90] Xin H. Wang F. Li Y. Lu Q. E. Cheung W. L. Zhang Y. (2017). Secondary release of exosomes from astrocytes contributes to the increase in neural plasticity and improvement of functional recovery after stroke in rats treated with exosomes Harvested From MicroRNA 133b-Overexpressing Multipotent Mesenchymal Stromal Cells. *Cell Transplant.* 26 243–257. 10.3727/096368916X693031 27677799 PMC5303172

[B91] Xu L. Cao H. Xie Y. Zhang Y. Du M. Xu X. (2019). Exosome-shuttled miR-92b-3p from ischemic preconditioned astrocytes protects neurons against oxygen and glucose deprivation. *Brain Res.* 1717 66–73. 10.1016/j.brainres.2019.04.009 30986407

[B92] You Y. Borgmann K. Edara V. V. Stacy S. Ghorpade A. Ikezu T. (2020). Activated human astrocyte-derived extracellular vesicles modulate neuronal uptake, differentiation and firing. *J. Extracell. Vesicles* 9:1706801. 10.1080/20013078.2019.1706801 32002171 PMC6968484

[B93] Yu A. Zhang T. Duan H. Pan Y. Zhang X. Yang G. (2017). MiR-124 contributes to M2 polarization of microglia and confers brain inflammatory protection via the C/EBP-alpha pathway in intracerebral hemorrhage. *Immunol. Lett.* 182 1–11. 10.1016/j.imlet.2016.12.003 28025043

[B94] Zang J. Wu Y. Su X. Zhang T. Tang X. Ma D. (2020). Inhibition of PDE1-B by vinpocetine regulates microglial exosomes and polarization through enhancing autophagic flux for neuroprotection against ischemic stroke. *Front. Cell Dev. Biol.* 8:616590. 10.3389/fcell.2020.616590 33614626 PMC7889976

[B95] Zhang D. Cai G. Liu K. Zhuang Z. Jia K. Pei S. (2021). Microglia exosomal miRNA-137 attenuates ischemic brain injury through targeting Notch1. *Aging* 13 4079–4095. 10.18632/aging.202373 33461167 PMC7906161

[B96] Zhao C. Deng Y. He Y. Huang X. Wang C. Li W. (2021). Decreased level of exosomal miR-5121 released from microglia suppresses neurite outgrowth and synapse recovery of neurons following traumatic brain injury. *Neurotherapeutics* 18 1273–1294. 10.1007/s13311-020-00999-z 33475953 PMC8423926

[B97] Zhou M. Hu M. He S. Li B. Liu C. Min J. (2018). Effects of RSC96 schwann cell-derived exosomes on proliferation, senescence, and apoptosis of dorsal root ganglion cells *in vitro*. *Med. Sci. Monit.* 24 7841–7849. 10.12659/MSM.909509 30387453 PMC6228118

